# Low limb prostheses and complex human prosthetic interaction: A systematic literature review

**DOI:** 10.3389/frobt.2023.1032748

**Published:** 2023-02-13

**Authors:** Adan Domínguez-Ruiz, Edgar Omar López-Caudana, Esther Lugo-González, Francisco Javier Espinosa-García, Rocío Ambrocio-Delgado, Ulises D. García, Ricardo López-Gutiérrez, Mariel Alfaro-Ponce, Pedro Ponce

**Affiliations:** ^1^ Institute for the Future of Education, Tecnologico de Monterrey, Mexico City, México; ^2^ Instituto de Electrónica y Mecatrónica, Universidad Tecnológica de la Mixteca, Huajuapan de León, Oaxaca, México; ^3^ División de Estudios de Posgrado, Universidad Tecnológica de la Mixteca, Huajuapan de León, Oaxaca, México; ^4^ CONACYT-CINVESTAV, Av. Instituto Politécnico Nacional 2508, col. San Pedro Zacatenco, Ciudad deMéxico, México; ^5^ Institute of Advanced Materials for Sustainable Manufacturing, Tecnologico de Monterrey, Mexico City, México

**Keywords:** powered prostheses, complex systems, human-prosthetic interaction, control systems, artificial intelligence, lower limb amputation, innovative education, higher education

## Abstract

A few years ago, powered prostheses triggered new technological advances in diverse areas such as mobility, comfort, and design, which have been essential to improving the quality of life of individuals with lower limb disability. The human body is a complex system involving mental and physical health, meaning a dependant relationship between its organs and lifestyle. The elements used in the design of these prostheses are critical and related to lower limb amputation level, user morphology and human-prosthetic interaction. Hence, several technologies have been employed to accomplish the end user’s needs, for example, advanced materials, control systems, electronics, energy management, signal processing, and artificial intelligence. This paper presents a systematic literature review on such technologies, to identify the latest advances, challenges, and opportunities in developing lower limb prostheses with the analysis on the most significant papers. Powered prostheses for walking in different terrains were illustrated and examined, with the kind of movement the device should perform by considering the electronics, automatic control, and energy efficiency. Results show a lack of a specific and generalised structure to be followed by new developments, gaps in energy management and improved smoother patient interaction. Additionally, Human Prosthetic Interaction (HPI) is a term introduced in this paper since no other research has integrated this interaction in communication between the artificial limb and the end-user. The main goal of this paper is to provide, with the found evidence, a set of steps and components to be followed by new researchers and experts looking to improve knowledge in this field.

## 1 Introduction

Lower extremity amputation is the most common type of amputation in the world, causing a high physical and physiological impact on all patients as the lower extremities carry weight and control locomotion (Ebnezar et al., 2017). Even though amputations can occur at any stage of life, recent statistics (WHO, 2022) indicate that the three groups with higher prevalence of amputation are between 45 and 59 years of age. Also, it is important to note that amputation can be the result of not only an accident but also of disease or congenital deficiency. By 2019, 57.7 million people were registered to have undergone a lower limb amputation (McDonald et al., 2021).

Amputations are performed in patients looking not only to save their lives but also to allow them retain the same mobility/functionality as before (Espinoza and Garcia, 2014). A specialist determines the level of amputation to be performed, looking to keep the patient’s life safe and a functional limb for prosthesis; for the lower limb, the level of amputation can be classified as follows (Ebnezar et al., 2017):

•**Hemipelvectomy or trans-pelvic amputation:** Since it is proximal to the core of the body, it can affect a person the most to be adapted in their previous lives.
•**Above the knee or transfemoral amputation:** At the femur level.
•**Knee-level amputation.**
•**Below the knee or transtibial amputation:** between the knee and the ankle.
•**Partial foot or ankle amputation.**

To determine whether a patient is suitable for amputation, a specialist and, in some scenarios, software, such as the Amputee Mobility Predictor, must consider two factors (Mduzana et al., 2018): first, if the candidate has some pre-existing conditions that can reduce the probability of adaptation to a prosthetic device; second, if the prosthetic device could benefit the patient’s life.

Lower limb prostheses focus on helping recovery from lower-body locomotion, such as walking or running. To recreate this movement with a prosthesis, it is imperative to understand the biomechanics of steps.

To take a single step during walking, the human body must recognise the ground beneath them and decide how to move. In the case of an artificial limb, this information will be used to get the required level of kinematics prediction and movements to be performed. In a regular walk, as in humans, there is no need to think about how body balance works; the body does it by itself. However, in prosthetic development, the artificial lower limb must work together to obtain the correct balance without damaging the residual limbs (Rodrigues et al., 2021). One step is divided into two stages:

•**Stand:** Defined as the moment measured by the Ground Reaction Force equal to the weight of a person or during walking when this is greater than 50 N (Hunt et al., 2021). The step-demarcation of the ankle defines the limits of balance during this position, and the weight between the two human hemispheres must be balanced (Rodrigues et al., 2021).
•**Swing:** Starting when the foot leaves the ground and ending when touching it again, making it 40% of the gait cycle (Filho et al., 2021).

Most studies focus on the following areas of the study of movement: locomotion change, control of gait speed, and control of the direct ankle or knee joint. In addition, research focuses on improving control, giving the patient a more natural feeling of control that is easier to learn and intuitive, to lower the previously mentioned low usage percentage. A prosthesis is designed according to the activity performed by the patient. For this research, two main classifications of prostheses have been identified.

1. **Passive:** Movements performed by the prosthetic system are created by using an external force (Maat et al., 2018).
  •Aesthetical: Provides the appearance of the organic limb with no extra functionality.
  •Tool: Device adapted to perform specific movements according to the desired activity.
2. **Active:** Defined as prostheses with external power and movement provided by actuators, offering high performance and functionality, at the cost of complexity (Windrich et al., 2016).

The development of active prostheses is a growing field of research, leading to the design of new and robust control systems and methods of interaction between artificial limbs and human intention. The main contributions of this study to the scientific community regarding active prostheses are the following:

•Definition of the term “human–prosthetic interaction”: Definition of a concept to be used in future literature to describe methods in which humans and artificial, active limbs communicate.
•**A view of trending technologies used in lower-limb bionics technologies**: How human interfaces can affect the movements of a patient and the control methodologies used to help a patient perform daily activities.
•**The research of a control structure and a design scheme for the design of new lower-limb prosthetic systems:** Researchers should follow the proposed structure as a generalised guide for new technologies developed in the field, giving the option to focus on the improvement of one of the subsystems to contribute as a whole.
•**Define the challenges and future direction of the technology:** Highlight the future work from different works being carried out by authors around the world. The current limitations and what needs to be improved.

**FIGURE 1:**
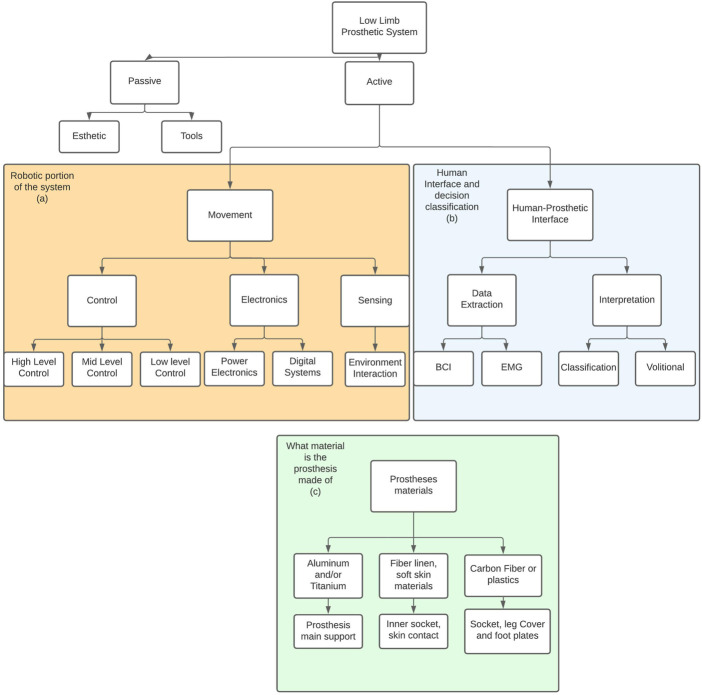
Lower-limb prosthetic system: showing the structure of the document and the subsections for each technology involved. **(A)** Shows the digital systems used on any active prosthesis, including sensing capabilities for devices with environment interaction. **(B)** Sensors used for constant communication with the patients. **(C)** Common materials used in prosthesis development.

Figure 1, shows the general classification of technologies employed in the development of lower-limb prosthetic devices. Used as a base for the structure of the manuscript and describing active prostheses, Figure 1A shows the digital systems of intelligent active prostheses and Figure 1B the interaction between the patient and the robotic device, while describing a short analysis on the materials used, as shown in Figure 1C. Section 2 presents the conducted systematic literature review methodology (protocol, search, selection, and revision). Section 3 presents the elements of the lower limb prosthetic device, the human–prosthetic interfaces, and the elements that allow the correct movement of the prosthesis challenges and trends. Section 4 discusses the progress and application of the current technologies in a prosthetic system. Section 5 presents the trends in the areas of control, human signal reading, and environment interaction. Finally, Section 6 presents the conclusion.

## 2 Search methodology

At looking at the human body as a complex system and the interaction with artificial limbs in an environment where daily activities are involved with mental and physical wellness, it is important to follow a systemic approach (Baena-Rojas et al., 2022). For this analysis, a systematic literature review (SLR) method is employed, guided by Kitchenham and Charters. (2007), consisting of three phases: planning, conducting, and reporting, finally showing the results.

### 2.1 Research question

Considering the objective of this review, a research question was defined, followed by the subquestions shown in Table 1 to assist in the analysis of the literature and detect key components of intelligent control, prosthesis types and trends, or future work.

**TABLE 1:** Research questions and expected answers based on research.

Questions	Type of answer sought
How many studies are in the Scopus and WoS databases between 2016 and 2021 and what is their design?	No. of articles in Scopus
	No. of articles in Web of Science
	Prosthetic design structure
Which journals have the greatest number of publications on this topic?	Q1, Q2, Q3, or Q4, ESCI, No rank
How are the studies classified?	Hardware
	Computer science
	Biomechanics
	Robotics
What are the trends and topics addressed by the articles?	Energy management
	Hardware improvements
	Control techniques
	Sensors and signal reading improvements

These questions lead to the use of strings and search terms in Table 2, with the selection criteria in Table 3, applied on September 20, 2021, in two scientific citation databases, Web of Science and Scopus.

**TABLE 2:** Strings created on the Scopus and WoS databases.

Search strings in Scopus	Search string in WoS
KEY (brain AND computer AND interfaces)	TS = (biomechanics OR myoelectric OR eeg
AND [biomechanics OR eeg OR myoelectric OR (body AND signals)]	OR (Body Signals) OR (haptic feedback))
OR (haptic AND feedback) AND (prosth*)	AND TS = (Brain–Computer Interfaces) AND TS = (Prosthe*)

**TABLE 3:** Inclusion/exclusion criteria for selected articles.

Inclusion criteria	Exclusion criteria
Studies indexed in the Scopus and WoS databases	Conference, early access, or proceeding papers
Articles published between 2016 and 2021	Articles from emergent sources
Articles related to robotics, computer science or control	Articles not published between 2016 and 2021
English or Spanish language	Articles published in Russian
Field categories: physics, engineering, materials neuroscience, computer science and maths, and multidisciplinary	

**RQ.** What is the state of the art in intelligent lower-limb prosthetic devices and their design structure?

**FIGURE 2:**
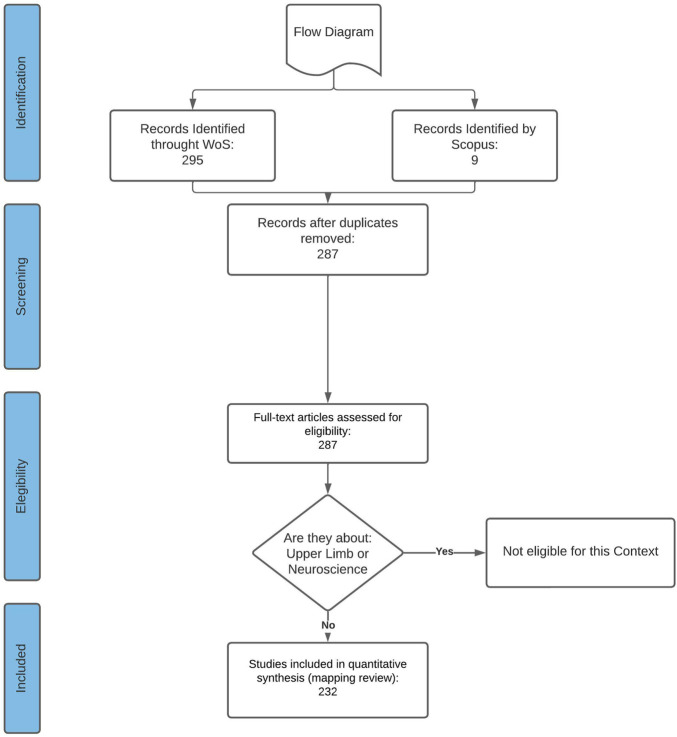
Selection process based on the PRISMA methodology, and the specific search string and inclusion/exclusion criteria can be seen in Tables 2, 3.

By selecting 194 relevant articles using the PRISMA methodology criteria given in Figure 2, the systematic review is structured to be applied by other researchers carrying out an automated string search. Table 4 shows that the journal identified with the highest number of articles published was IEEE Transactions on Neural Systems and Rehabilitation Engineering and Frontiers, which, by the use of different specific issues, provides a wide variety of data belonging to this area.

**TABLE 4:** Journals with at least three articles.

Articles	Database	Source title	Quartile	Country
43	Scopus	IEEE Transactions on Neural Systems and Rehabilitation Engineering	Q1	The United States
19	Scopus	Journal of Neural Engineering and Rehabilitation Engineering	Q1	The United Kingdom
7	WoS	Frontiers in Neuroscience	Q2	Switzerland
7	WoS	Biomedical Signal Processing and Control	Q1	The Netherlands
6	Scopus	IEEE Access	Q1	The United States
4	Scopus	Sensors	Q2	Switzerland
4	Scopus	IEEE Transactions on Biomedical Engineering	Q1	The United States
4	WoS	Journal of Neuroengineering and Rehabilitation	Q1	The United Kingdom
3	Scopus	Frontiers in Neurorobotics	Q2	Switzerland

## 3 Prosthetic elements

**FIGURE 3:**
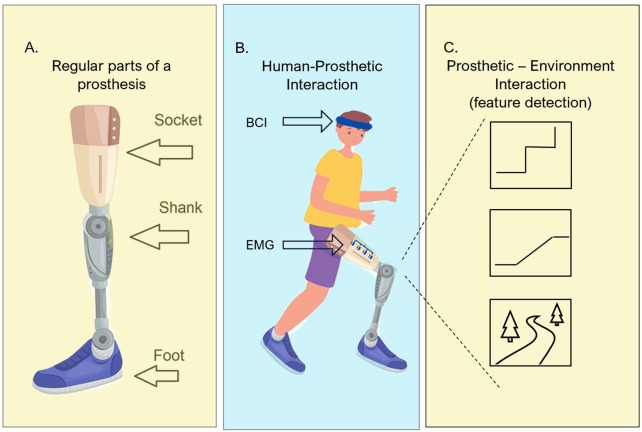
Parts of lower-limb prosthetic system (suspension and liner are not shown). **(A)** shows the physical components of general lower-limb prosthesis, where the shank is separated by knee artificial joint and by ankle joint. **(B)** How the communication components need to interact with the user, a small sketch of brain–computer interface (BCI) and electromyogram (EMG) sensor position is shown. **(C)** With embedded sensors, the prosthetic device needs to be aware and detect the features of the surroundings.

Once a patient is found suitable for a prosthetic device, the device is tailor-made according to the patient’s needs. Figure 3A a shows a visual representation of the parts on a general lower limb prosthetic device (Ebnezar et al., 2017).

•**Socket**: Crucial for prosthetic performance since it encapsulates the residual limb or stump, offers comfortability, and disperses the body’s weight into different pressure tolerance areas to create a distributed weight on the residual limb for different activities during the day (O’Keeffe and Rout, 2019).
•**Suspension**: Part used to keep the prosthesis attached to the body.
•**Liner**: The removable inner part of the socket is used to provide a soft feeling to the skin.
•**Shank**: The body of the prosthesis, which is usually made of aluminium, titanium, or carbon fibre, works as the main body of the prosthesis.
•**Foot or end-point**: simulates the foot of a human and is to be used for support and shock absorption during standing or walking.

This manuscript shows a representation of two extra components that must be present in active intelligent prosthetic devices: a human–prosthetic interaction layer (Figure 3B) and a method of interaction with the environment to predict required movements (Figure 3C).

**FIGURE 4:**
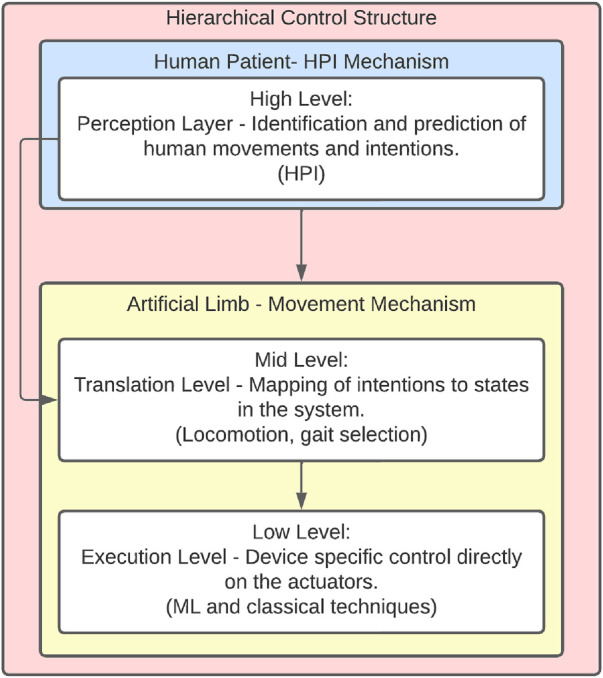
Generalised hierarchical structure of control. Proposed framework adapted from Tucker et al. (2015). HPU module and pattern recognition define locomotion mode and gait selection. Low level direct control the joints on the device.

To be considered a smart prosthetic device, these components must be able to provide movement independently; therefore, research has focused on controlling brushless DC motors or pneumatic and hydraulic actuators. A control structure shown in Figure 4 is used, indicating the requirement of two mechanisms consisting of an interface for human–prosthesis interaction and an artificial movement method that is coupled with natural human reactions.

### 3.1 Human–prosthetic interaction

Based on this review, there were some terms found that refer to active prosthetic devices acting on human orders. Terms such as human–mechatronics interaction, by Clites et al. (2018), define a relationship between human and machine, or human–robot interaction, according to Sheridan (2016), and focus the concept on four areas: Human supervisory control of robots in routine operations, remote control of autonomous vehicles, automated vehicles with human passengers, and social robotics. However, the definitions are very extensive, and a new term was necessary to refer to the communication between an artificial limb and a human user; this study proposes the term human-prosthetic interaction (HPI). Depending on the level of the interaction, the user might feel like using their own limb.

For a successful interaction, the extraction of data from the human body and a method of interpretation are required. Two techniques are used in the literature to extract data on the intention of movement; Table 5 shows an overview of their advantages and disadvantages.

1. **Electromyogram signals:** Described as a technique to evaluate and record electrical activity produced by muscles (Cabral et al., 2018). These can be of two types: surface (sEMG) and intramuscular electromyography (iEMG).
  •iEMG: Signals are detected with special needles or wires inserted directly into specific muscles. This technology is used on prostheses attached surgically to the body. However, it is not commonly used because it is highly invasive (Merletti and Farina, 2009).
  •sEMG: Signals are detected with sensors placed on the muscle surface, usually with two or more electrodes since it measures the electrical difference between activated muscle and a reference point (Cabral et al., 2018). Several studies (Luu et al., 2017; Yu et al., 2017; Su et al., 2019; Peng et al., 2020b) use the described method of interaction due to its almost instant response, non-invasive technology, and ease of use. However, the disadvantage of this method is the noise produced by close muscles (Nieveen et al., 2020), so it requires an analysis of the residual limb activation when performing different activities (Gupta and Agarwal, 2019; Su et al., 2019; Wang et al., 2022). To overcome this limitation, Clites et al. (2018) used sEMF in combination with a surgery method called agonist–antagonist myoneural interface (AMI) amputation, using sensory feedback, making the signal clearer for use.
2. **Brain–Computer Interfaces**: The technique used to record brain activity to determine a desire to move, control, or interact with the environment. Most BCIs are based on electroencephalography (EEG) signals; EEG records the brain’s electrical activity and is popular in the BCI due to its portability, low cost, and spatiotemporal response, allowing the BCI to act as a real-time projection of brain activity during multiple actions. (Shafiul Hasan et al., 2020). According to the purpose of the BCI, the EEG electrodes are selected taking into account the brain lobes associated with the movement task; also, EEG signals are classified by their rhythmic activity into mu, delta, theta, alpha, beta, and gamma based on signal frequencies ranging from 0.1 Hz to more than 100 Hz. McFarland (2000) found an association between movement and motor imagery with mu and beta rhythm resynchronization; however, Chen et al. (2021); Wriessnegger et al. (2018) worked with the different frequencies mentioned to detect activities such as playing tennis or squeezing a ball, and depending on the skills, the activity varied. The HPI method has been widely used in different works (Lu et al., 2017; Su et al., 2019; Yokoyama et al., 2021) due to the high precision that might be obtained by volitional control or motor imagery.
3. **Intention prediction based on environment interaction**: A third option to be used by prosthetic systems is for sensors to interact with the external environment, and based on it, the next movements are predicted; however, IMU, force plates and force sensors, and cameras are some of the most commonly used equipment, and a more detailed review of these systems is covered in the Sensing subsystem (Section 3.2.1).

**TABLE 5:** Advantages and disadvantages of methods for reading human movement intentions.

Human–robotic interface	Pros	Cons
Neurological signal control (motor imagery)	High movement accuracy	High computational cost
	Not much training required	High monetary cost on the hardware required
		Uncomfortable caps or invasive technology required
Myoelectrical signal Control	Fast response	Depends on the level of amputation
	Requires simple interface	Requires more training
	More economical	Movements might be limited
	Low computational cost	Much noise on the signal reading

Data must be interpreted to perform the user-desired action. First, the obtained signals must be preprocessed to remove any noise coming from the position of the electrodes, cables, etc. Then, machine learning techniques (feature extraction, feature selection, and classification) are employed to detect patterns related to the movement intention in these biopotentials (Table 6).

**TABLE 6:** Machine learning techniques; to get patterns and predict intentions from the user for lower-limb prosthetic control.

	ML techniques					
Author	Feature extraction	Feature selection	Classifier	Validation technique	Input data	Reported accuracy
Toledo-Pérez et al. (2019)	—	Principal component analysis (PCA)	Support vector machine (SVM)	—	EMG	95%–100%
Su et al. (2019)	—	—	Convolutional neural network (CNN)	Cross-validation	Motion intention from IMUs	94.15% for the able-bodied and 89.23% for amputees
Gupta and Agarwal. (2019)	—	ANOVA, *ρ*-value <0.05	SVM, linear discriminant analysis (LDA), and neural network (NN)	10-fold cross validation	EMG	96.83 ± 0.28%, 97.45 ± 0.32%, and 97.61 ± 0.22% respectively
Sattar et al. (2021)	Signal mean (SM), variance (*σ*), skewness (SSK), kurtosis (SK), slope (SS), waveform length (WL), mean absolute value (MAV), root mean square (RMS), Willison amplitude (WAMP), and Zero Crossing (ZC)	—	k-nearest neighbours (KNN)	10-fold cross validation	EMG	Offline 95.8% and 68.1%, real-time 91.9% and 60.1% for healthy and amputated subjects, respectively
Brantley. (2019)	Power spectral density (PSD) and total power in the *θ*, *α*, *β*, and *γ* bands	PCA	SVM	10-fold cross validation	EEG	80% in offline decoding
Wang et al. (2022)	—	—	Cascade classifiers with a gait phase dependence	—	EMG and IMU	99.13% and 99.39% for the standing and swing phases, respectively
Meng et al. (2021)	RMS	—	SVM	5-fold cross validation	EMG and linear acceleration data of lower limb	98%

To take lower-limb active prostheses as a separated device from the human body shows higher cognitional load on the users and acts as a limitation for devices with only one-way communication (Raspopovic et al., 2021). As a complement, some researchers are working on feedback to enable communication from the prosthesis to the human cognition system, with the hypothesis of increasing accuracy and improving control. The use of vibrotactile actuators Shi et al. (2019); Chen et al. (2016) allows the residual limbs to feel what the actual prosthesis is touching. Surgery performed to restore the muscle relationship between agonist and antagonist muscles (AMI) (Srinivasan et al., 2020) has shown higher ankle control, reshaping the nerves to adapt an electrode (Leventhal and Durand, 2002) has shown higher equilibrium capabilities, and electrode insertion into peripheral nerves (Badia et al., 2011) has provided confidence in the users by avoiding falling probabilities to walk on uneven terrains (Raspopovic et al., 2021), which restores the muscle relationship to improve motor control Srinivasan et al. (2020).

### 3.2 Movement development of a prosthetic device

After obtaining and classifying the desired movement, the performance of the actual movement starts. For this, the prosthetic device must have a sensor subsystem in charge of interacting with the environment, electronic components consisting of power and a digital interface, and an automatic control method.

#### 3.2.1 Sensing subsystem

This subsystem works both as a method to collect data from the environment to be used in the calibration and control of the prosthetic device and to evaluate its level of performance. One of the most common sensors is the inertial measurement unit (IMU), a device capable of measuring the angular rate, acceleration, and magnetic field surrounding the system. The device IMUs can help optimize the user fitting and alignment and track changes in gait speed over time *via* different algorithms (Bastas et al., 2018). The sensor can help track and estimate the locomotion trajectory on the knee or ankle depending on the type of terrain where the user is walking (Chang et al., 2019; Su et al., 2019; Elery et al., 2020; Gaetani et al., 2020; Pi et al., 2020; Khademi and Simon, 2021; Lee et al., 2021).

Also used as complementary data for other measures is the ground reaction force (GRF) exerted by the ground on a body at contact. By Newton’s third law, when a person is standing, the GRF will be weight; however, acceleration forces change when moving, usually when working together with a treadmill with plaques or force sensors, such as flexiforce (Peng et al., 2020b) and M3715C (Gao et al., 2021), on the feet of users (Jiang et al., 2019; Lee et al., 2021) to detect torque changes with gait variations. GRF has been used to analyse the biomechanics of a non-amputee and compare it with how locomotion changes after amputation when walking with different prostheses (Chang et al., 2019; Jiang et al., 2019; Zhang et al., 2019; Peng et al., 2020b; Mendez et al., 2020; Pi et al., 2020; Gao et al., 2021; Lee et al., 2021; Leestma et al., 2021).

Calculating muscle effort has also been described using force analysis with inverse or forward dynamics and using EMG or centre and point of mass models (Chiu et al., 2020). With these data, metabolic usage can be obtained to compare the use of different prostheses and how degrees of freedom (DoF) and control help the patients (Zhao et al., 2019; Elery et al., 2020; Kim et al., 2020; Hunt et al., 2021).

A big improvement found on the transtibial prosthesis carried out by Clites et al. (2018) and others was the capability to get neural feedback, in their words, like the prosthesis was alive. To create similar behaviour, with no need of an AMI surgery, is to provide the prosthetic system with the ability to see their surroundings, improving the track of motion and kinematics of steps at different speeds; cameras and depth cameras have been used (Jiang et al., 2019; Zhang et al., 2019; Dimitrov et al., 2020). Encoders and current sensors are closely related to methods of angle control and energy management (Bartlett et al., 2021; 2019; Chiu et al., 2020; Mendez et al., 2020; Keemink et al., 2021), and the same power consumption has been used as a way to measure the performance of control methodologies to minimize it Dong et al. (2020); Jiang et al. (2019); Khademi and Simon (2021); Sutawika et al. (2021) since batteries can cause an increase in the cost and weight of a prosthetic device, making it harder to use for the patient on a regular basis. However, power management comes with an issue; control methodologies can only reduce consumption by tweaking the parameters of the control algorithms, but actuators may be physically modified to find the best performance at a lower energy cost.

#### 3.2.2 Electronic subsystem

The electronics component is composed of an external power supply, its electronic power stage, a digital embedded system, and actuators.

For all the parts involved, a power supply is required when not tested in a controlled laboratory environment; for most microelectronic systems, the energy consumption can range from 3.3 V to 10 V, depending on the internal capabilities. However, actuators require high torque ( > 80 Nm) (Sup et al., 2008), which comes with high current demand, meaning high power in the range of 12–200 W (Dong et al., 2020; Zagoya-López et al., 2021), depending on the number of motors being used and creating the requirement of using an isolated energy source for them.

A power driver must be used for communication to isolate the energy used in the controller and actuators. For most of these designs, market-available hardware has been used, and Gao et al. (2021) and Pi et al. (2020) used an Elmo Driver at the knee and ankle level, or Elery et al. (2020) used the Solo God Driver. In the case of electronic actuators, the most widely used motor is brushless DC motors, showing good performance, short response time with high torque, and low energy consumption compared with the torque they can generate (Cheung, 2002). The most used motor found during the review is the Maxon RE40 DC motor with no gearbox (Dong et al., 2020; Pi et al., 2020) or EC-30 (Zagoya-López et al., 2021) and the Elmo servo motors (Gao et al., 2021). As previously discussed, the power supply must be separated, using 48 V DC power supply, either directly mounted on the prostheses itself (Cherelle et al., 2017; Dong et al., 2020; Ottobock, 2021) or even connected on a continuous energy source when still on development (Kim and Collins, 2017; Jiang et al., 2019; Elery et al., 2020; Kim et al., 2020), and a smaller lithium battery (Azocar et al., 2020; Dong et al., 2020) 5–7 V for the smaller electronics.

**FIGURE 5:**
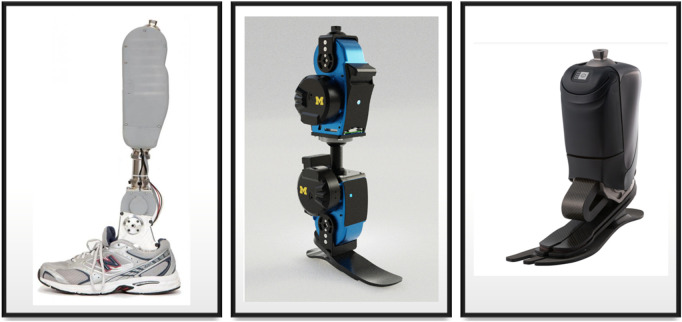
From left to right: Ottobock prosthetic ankle Ottobock (2021), Vanderbilt Shultz et al. (2015), and OpenSource Leg project Azocar et al. (2020).

Finally, a digital system processor or microcontroller is required to receive, interpret, and perform actions. Bartlett et al. (2021); Chang et al. (2019) used an interface between the prosthetic system and a computer to perform easier debugging. However, for more advanced systems such as the ones in Figure 5, an embedded microcontroller or processor is required for online sensing and control.

#### 3.2.3 Lower-limb biomechanics control

To define how a prosthetic device moves in combination with the user, a series of angles must be performed on the actuators using a control method. Different control schemes can be used, such as direct volitional, path tracking, motion, and impedance control.

The mid-level control is described as a transition level, as referenced in Figure 4, where the HPI signals are used as input to perform a mapping function of the human intentions to states in the system. Locomotion and gait are the common transition schemes based on FSMs, which give low-level direct joint control as output. Error minimisation control techniques are then used to perform these actions.

•**Locomotion**: Described in this context as the ability of a human to move from one place to another. For lower limb prostheses, six different locomotion modes have been found (Huang et al., 2011): level-ground walking, stepping over an obstacle, stair ascent, stair descent, ramp ascent, and ramp descent. The intention recognition and smooth transitions between them have been a work of research among different studies (Su et al., 2019; Zhang et al., 2019; Peng et al., 2020b; Pi et al., 2020; Khademi and Simon, 2021; Lee et al., 2021; Leestma et al., 2021). One of the most used schemes is direct volition control, which uses EMG or EEG signals to perform a volitional intention; in this case, the methodology used tries to predict behaviour.
•**Gait**: Refers to the movements performed inside the locomotion, the series of steps to perform an actual step in any locomotion type, usually composed of the swing and stance phases, with the transition between them, such as “heel on the ground, heel off the ground, toe on the ground, and toe off the ground” (Filho et al., 2021). These movements and the speed depending on the user’s intention have been tracked by BCIs, EMGs, and IMUs to get a more natural feeling of walking in different terrains (Gao et al., 2021), but this type of research uses Finite State Machines (FSMs) to focus on the whole trajectory tracking to minimise the studies on the dynamics of the movement (Mai and Commuri, 2016; Whitmore et al., 2016; Cao et al., 2018; Adamczyk, 2020; Peng et al., 2020a; Mendez et al., 2020; Shafiul Hasan et al., 2020; Sutawika et al., 2021; Welker et al., 2021; Yokoyama et al., 2021). Since the movement is a whole complex trajectory, instead of specific points to be reached by a single actuator, the schemes that are used must be robust against disturbances, and, in the case of multiple actuators, actions must be performed at coordinated times. Coordinated motion control, where joints are connected to each other and a range of motion is determined, could be used to avoid unwanted muscle activity; path tracking control determines a path to follow, adapting to any disturbance in the system (Hernandez and Yu, 2021).
•**Direct joints**: Taking the locomotion types and gait phases more accurately, this type of control focuses on how the actuators need to move independently to get specific angles, using an impedance/admittance control scheme. This scheme can be defined as a method to control the relationship between angular velocity and torque (Hernandez and Yu, 2021). When a user travels on different surfaces (Chang et al., 2019), the method allows small changes to occur in the swing phase of the gait, minimising energy consumption (Dong et al., 2020) by controlling the exact torque required in the joints to maintain balance in the human body. Related, the option to control more than a single degree of freedom to improve dynamic balance on real-world terrain (Kim and Collins, 2017; Huang and Huang, 2019; Dimitrov et al., 2020; Harper et al., 2020; Kim et al., 2020; De Vree and Carloni, 2021).

**FIGURE 6:**
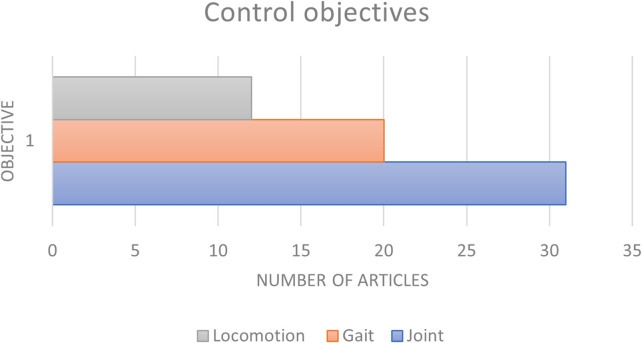
Articles divided as per the different control technologies identified, such as locomotion changes, gait or trajectory of swing tracking, and direct joint control.

The number of articles categorised by the objective of the control used, as described, is shown in Figure 6, where direct joint control is found to be the most common since it can help create precise movements for specific areas.

The PID controller (proportional–integral–derivative) was the most implemented in the literature. (Cherelle et al., 2017; Dong et al., 2020; Widhiada et al., 2020; Xie et al., 2020; Hongsheng et al., 2021), controlling power and torque directly on ankle actuators or following the gait trajectory by using torque and power consumption as the controller’s outputs(Sutawika et al., 2021). In the mentioned works, the advantages shown were low response time and steady-state error zero, making it helpful in controlling both fast and slow process variables.

A proportional derivative (PD) controller has the advantage of being easy to stabilise due to low dampening at the tracking set-point and the disadvantage of amplifying high-frequency noise, although it is not recommended for slow-moving process variables. The proportional integral (PI) controller has the advantage of no steady-state error but the disadvantage of a narrower range of stability and wind-up (Effiong and Obot, 2018), even though the type of control will depend on the activity to be controlled.

The PD controller is used in the classification of motion intention and the prediction of the trajectory (Gao et al., 2021), with less than 0.1 radians of error in detecting the expected gait before reaching the desired change based on the difference in terrain (slopes and stairs). On the other hand, it has been used in hydraulic actuators due to the PD controller’s response type Bartlett et al. (2019). The derivative value helps predict the values, with softer changes, which is very helpful for the discussed application. Lastly, proportional (P) controllers, even when simpler, mean that fewer values need to be tweaked, trying to control direct joint angles while performing stand and swing phases of walking (Hunt et al., 2021). The ankle stiffness was adapted to follow swing motion by using a camera system to track the trajectories of a step, using values from a P controller on (Dimitrov et al., 2020).

#### 3.2.4 Machine learning control modelling

**FIGURE 7:**
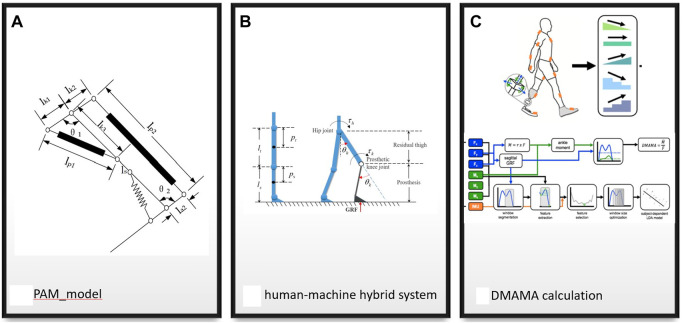
**(A)** Pneumatic artificial muscle structure: shows angles of movement and length of the bars. **(B)** Flexible prosthetic knee composition inertia and ground force reaction matrices give torque and angle. **(C)** Dynamic mean ankle movement arm different force and momentum values obtained by load cells are used as input for ML algorithms to classify different terrains.

Machine learning techniques were primarily used to get patterns and predict intentions from the user, as seen in Table 6. However, in locomotion control, movements were first classified in 2017 (Chmura et al., 2017), but more studies were carried out from in 2019 as a method to predict locomotion modes (Su et al., 2019; Peng et al., 2020a; Khademi and Simon, 2021) and obtain movement dynamics (Li et al., 2021) with less computational real-time power. This was combined with the classical control of artificial joints to obtain almost instantaneous reactions to the environmental characteristics recognised by the system (Zhang et al., 2019). Classical linear control methods require a high understanding of the dynamics of the mechanical components, such as with Hongsheng et al. (2021) in Figure 7A, where the pneumatic actuators are controlled by a series of highly complex equations with changes depending on the expected movement and the lengths of the bars, creating the problem of a great method of control for a very specific task, which is not the case for everyday use. By using a hybrid model seen in Figure 7B, combining the inertia matrix (*M*(*θ*), Coriolis and centripetal values (*C*(*θ*)), gravitational force vector (*G*(*θ*)), and a fuzzy neural network to estimate the time estimation values, Peng et al. (2020a) were capable of calculating the required torque and angle in the knee movement capable of handling disturbances affecting the swing trajectory.

Complexity increases widely when adding degrees of freedom; however, as seen with Leestma et al. (2021) and Jiang et al. (2019), values obtained from the sensors used, as seen in Figure 7C, can be used as input values in a machine learning algorithm such as in neural networks (Keleş and Yucesoy, 2020) to control the kinematics of every step; however, the limitations of Leestma et al. (2021) reside on the test being performed, used in very controlled level ground scenarios with some speed variations.

Finally, machine learning has also been used as a simulation method in which non-amputee walks have been recorded to compare them with the values collected from movement with a prosthesis, capable of obtaining the constraints and parameters of a healthy human walk in different terrains without an explicit model (Welker et al., 2021).

## 4 Discussions

**FIGURE 8:**
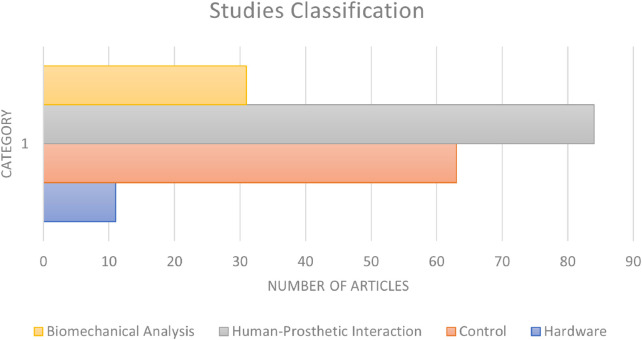
Categories of technologies identified in the literature. For this purpose only, the articles that made a contribution in lower-limb human prostheses, or human–prosthetic interaction (HPI), were counted.

Regarding the development of hardware and software for a lower-limb prosthetic device, the structure of Figure 4 was determined to work best, dividing the system into HPI and movement mechanisms. To develop a prosthetic system, different areas of study must be combined, including medical and engineering expertise, as shown in Figure 8, which shows how the literature is divided into biomechanical analysis, control, hardware development, and understanding human signals.

A major focus must be on the biomedical area, combining the expertise of engineers in mechanical work with that of healthcare workers to understand the basics and more advanced techniques of human locomotion, including balance techniques and muscle reactions to different external disturbances. Section 3.2.3 describes the method in which a prosthesis can be controlled, and Section 3.1 can progress the understanding of the patterns of intentions of human signals.

Progress on technologies used in communication between humans and prostheses has shown that a new concept is required, proposed in this paper as HPI, used in this context as how a user sends the desired intentions for the artificial limb to perform the actual movement. It was found through the literature that even though EMG technology has been in operation for a longer time, only 25 articles use this method as an HPI method or perform further signal analysis research. EEG technology has been shown in 57 articles, showing that there are still many opportunities for research in this field. The data of EEG are more complex and require more computational cost and the use of caps or invasive technology. However, it provides accurate movements once decoded, and the actual process of think-to-move is more intuitive than those of EMG-controlled devices. Even with a fast response, the latter is more economical and, with simpler human interfaces, depending on the level of amputation, will require non-orthodox movements of the residual muscles to reach a simple desired location. Using both has improved the accuracy and natural feeling of the expected movements (Hu et al., 2018; Ruhunage et al., 2019).

Similar methodologies were found in the experiments using the HPIs discussed. By using a similar number of amputated and non-amputated subjects (Twardowski et al., 2019) to perform the same movements and comparing the results, experimentation was possible, as with Idowu et al. (2021) and Ruhunage et al. (2019), which used only amputees and healthy subjects, respectively. An institutional review board had to approve the study in any of these scenarios. The advantages and disadvantages of the different methods of reading human intentions are shown in Table 5. By using a different type of approach, volitional control/intent recognition, it is possible to lower the pressure on the pattern and motion recognition algorithms; however, as discussed in Redkar and Bhat G (2018) and Tucker et al. (2015), this method requires high precision and focus from the user’s site. Overcoming this issue requires a combination of volitional control on high-precision movements and pattern recognition for locomotion and gait changes. The output of this subsystem goes directly to the type of control in which knowledge of human locomotion is necessary from an engineering perspective to be able to follow the kinematics and trajectories of a single step in different scenarios.

The different techniques of control, as seen with the analysis, depend on the level of movements to be performed, either direct joint control or whole trajectory tracking. These values need to be tweaked based on a biomechanical analysis and specific activities to be performed. Machine learning techniques, on the other hand, are used to reduce the effort required to learn this exact dynamic model, providing a faster response, adaptation to unknown disturbances, and the use of less computational power to tweak certain parameters.

These control techniques depend on the hardware being used, requiring mechanical engineering expertise. The most used mechanisms are electromechanical actuators, using DC brushless motors such as Robodrive and Maxon (Elery et al., 2020; Pi et al., 2020), due to the benefits of power management, control properties, and working directly by electrical current. Using pneumatic muscles (Li et al., 2016; Mrazsko et al., 2020; Xie et al., 2020; Hongsheng et al., 2021) has advantages in the amount of force-to-weight ratio it can offer, providing results of performance similar to that of an organic leg, although lacking the ability to produce accurate movements and an interface between electrical-air action. Hydraulic actuators (Chmura et al., 2017; Cao et al., 2018; McGrath et al., 2018; Bartlett et al., 2019) are similar to pneumatic actuators, with the difference of using liquid instead of air. These allow for even higher peak power and lower foot-to-ground shocks. However, the cost increases, requires more maintenance in the actuators, and requires multiple additional components to get accurate desired positions. BiOM (Rouse et al., 2015) and Ottobock (Mosler, 2021) prostheses are some examples of market available advanced systems used for specific activities. Table 7 shows the characteristics of the models available in the current market, while Figure 5 shows a visual representation. Unfortunately, most of them are unavailable for researchers to use with their current controller technologies. To address this issue, different universities developed the open-source robotic leg project (Rouse et al., 2015), in which every researcher can get the files to create the same version of the prosthesis for further development in the area, giving them the opportunity to only focus on methods of controlling and improving the same hardware.

**TABLE 7:** Available prosthethic hardware on the market Lawson et al. (2014), Ottobock. (2021), Azocar et al. (2020).

	Vanderbilt	Ottobock	OpenSource Leg
Knee range of motion	120°	120°	120°
Ankle range of Motion	70°	45°	30°
Weight	5 kg	4.7 kg	3.7–4.2 kg
Cost (U.S. dollars)		40,000 to 120,000	10,000 to 30,000
Materials	Aluminium and carbon fibre	Wood, plastic, foam, and carbon fibre	Aluminium and carbon fibre
Extras	Transtibial (ankle) prosthesis is available as separate design	Different models can be selected variating the activity to be performed and level of amputation	Customizable for sensors and other actuators

Since the artificial limb is a device a patient uses, it must interact with the environment to take actions on its own, as a natural limb does it unconsciously, an activity performed by a sensing subsystem. IMU sensors are the most widely used in the literature to collect information from the environment, the movement of a non-amputated user to understand and study biomechanical data (Jiang et al., 2019), or directly in a prosthetic device for real-time control (Mendez et al., 2020). They are a versatile option for the benefits of price and size. The problem with this technology is that the papers shown do not explain how the data were gathered, and it is important to reliably extract the specific parameters of step-demarcation with existent algorithms for lower limb prosthetic users Bastas et al. (2018). GRF sensors have fewer problems with data gathering and analysis since they depend directly on the user’s weight and how they vary according to the terrain, locomotion type, and gait. This can be carried out by using two different sensors: plaques on the floor, which can gather more reliable data but can only be used in biomechanical analysis directly on treadmills or in highly controlled and built environments, or pressure sensors directly on the prosthetic foot (Gao et al., 2021). The benefit of this technology in biomechanical analysis is that it can detect anomalies or excessive residual muscle effort that could create future problems in a patient with a new prosthetic device. To measure the performance of the prosthetic device, camera motion tracking systems, muscle effort and metabolic usage calculations, and even the power consumption of the prosthesis have been carried out. Camera tracking systems can compare trajectories between different prosthetic systems being used and non-amputee walking; on different works, it has been shown to excel at identifying small changes and how terrain changes can variate variables in human biomechanics, such as torque, metabolic cost, and angles in the body (Zhang et al., 2019; Mendez et al., 2020; Xiu et al., 2022), The Vicon motion tracking system has been widely used in different occasions (Thomas, 2018; Dimitrov et al., 2020; Slade et al., 2021). Metabolic usage has been used as a method to compare the performance between different prostheses, just as with Jeon et al. (2022); Takahashi et al. (2015), where the use of active prostheses made the patients show less metabolic cost than using passive prostheses, or with Ingraham et al. (2018) and Askew et al. (2019), which demonstrated that the correct selection of a prosthesis could lead to less energy expended by the user; however, by increasing degrees of freedom, metabolic cost has shown little change since the isolation of specific factors could not be determined (Kim and Collins, 2017). Muscle effort, on the other hand, has been used to determine how the risk of damage to a patient’s residual limbs can be lowered (Bellmann et al., 2019; Elery et al., 2020; Hunt et al., 2021). Finally, power consumption has been the focus of research to reduce the amount of energy used to optimise the use of batteries in the prosthetic device (Sup et al., 2008; De Pauw et al., 2018) since larger batteries mean higher weight, so optimally, a prosthetic leg should provide a similar amount of steps taken by a regular human being every day [5,000–7,000 according to Berko et al. (2016)].

The limitations of the review are related to details on the EEG and EMG methodologies, including where electrodes must be located and the databases used. The electronics scope did not cover the specific microcontrollers and processors used and the programming language. Lastly, the review was carried out from an engineering perspective, which did not cover the institutions and procedures involved for an amputee to be selected as a candidate for a prosthetic device since it could vary depending on the country and economic capacities.

## 5 Future direction

The trends in the literature are divided into categories covering the future work for either the upper or lower limb, HPI, and hardware development. The analysis identified a gap in generalised machine learning models as the largest area of opportunity. This technique is widely used in the area of pattern recognition for EMG and BCI; however, as shown with Shafiul Hasan et al. (2020), real-time projection of brain activity is still being investigated, and the techniques require more work to identify parameters and increase processing speed. Closely related to body signals, the use of EMG or BCI has disadvantages, such as the quantity of training and limitations or failure to identify certain movements due to high noise in real-time situations. The results obtained by Hu et al. (2018) and Ruhunage et al. (2019) have shown that the use of both sources or even using EMG signals from different places can lower uncertainty and provide higher precision on intention classification. Used in combination with volitional control, all these methods can be used for either high-level control of the locomotion and gait selection or more dexterous control in crowded places or uneven terrains.

For locomotion prediction, as seen with Welker et al. (2021), humans can adapt to different techniques of manual selection, showing less than 8% in the error between expected versus actual position on ankle angle. An improvement can be seen with Peng et al. (2020a), in which by using autonomous locomotion prediction, the user does not have to emulate the same movement with another limb nor specify a manual change to the desired terrain. The results of these experiments show a high percentage of accuracy (more than 80% on most of the predictions), with the problem appearing when defining an initial certainty value of the possible next terrain, in which the manually selected values can increase detection errors (Stairs Down locomotion mode, with 67% of detection rate). To overcome the issue of prediction with only the previous and current states, machine learning and specifically deep reinforcement learning techniques for human locomotion have been used to improve detection based on repetitive learning Huang et al. (2011); Peng et al. (2020b); Meng et al. (2021), and using a combination of sensors to view the environment Zhang et al. (2019), it is possible to adapt to complex environments and motions to perform on uneven terrains (Song et al., 2021).

There is still a gap in neural networks or deep-reinforcement learning methods that work in conjunction with mathematical models of leg biomechanics to improve reaction time and locomotion prediction with no manual human intervention. The research focuses mainly on knee movement and ankles with 1 degree of freedom on lower-limb prostheses. Understanding that the balance of the human body uses techniques based on hip–ankle movements (Kuo et al., 1993) on more than one axis, research on two or three DoF could be a path to follow while keeping between limits in the weight and size of the whole prosthesis. Research on ankle movement is an emerging topic because of its capability to keep the balance of the human body by adapting to the terrain and the activity being performed. Although it is not feasible for an all-terrain prosthesis at this point, further studies are needed on uneven ground to adapt to the human leg.

Section 3.2.2 discusses the importance of energy consumption in prostheses. As a result, the task will demand less or more energy, and a prosthetic device that can perform complex movements will need the largest batteries, increasing the size, weight, and cost of these technologies. The works of Chiu et al. (2020), Cherelle et al. (2017), and Bartlett et al. (2021) show how future work requires a focus on the development of energy management techniques to minimise power usage to perform simple tasks. At this moment, prosthetic systems weigh between 2.27 kg (Xu et al., 2021) and 5 kg as shown in Table 7, while in terms of power, Ottobock devices, which have been identified as the longer running prosthesis, can run up to 8 h, compared with 13 h that healthy amputees walk in average, even when the exercise was minimised after the amputation Halsne et al. (2013); Diment et al. (2022). To achieve these improvements, it is necessary to develop design-specific actuators that could achieve peak performance at lower energetic cost, extend the usage time, and maintain balance in difficult areas to move.

Identification of surroundings in the prosthetic device and patient is a work in development in the literature; the usage of cameras and IMUs to detect uneven terrains and obstacles that could harm the user depends on the capacity of the processor being used and has a limitation on the weight of the whole system. When compared, autonomous vehicles, such as Tesla. (2022) and Mercedez. (2022), use similar sensors such as radars, lidars, IMUs, and cameras, leading to different advantages or disadvantages in being used on a small device. The results have created an opportunity for a search for guidelines on using these sensors on portable systems.

## 6 Conclusion

This systematic literature review establishes a prosthetic system’s structure and state of the art. Research focuses on improving the control used, energy consumption, and HPI accuracy. A design structure was proposed based on the parts involved in any new systems looking to be developed, and based on it, the structure of control was modified and proposed to be followed by new researchers in this area. This review contributes by following the proposed structure and focussing on the trends and gaps found in the literature.

In addition to a state-of-the-art review, the contribution of this article differs from that of previous articles by showing prosthetic systems as a whole, combining the HPI, environment interaction, and the electronics and digital systems. It is necessary to see prosthetic systems similar to natural limbs, constantly interacting with human intentions and the environment around them, which led us to define the term human–prosthetic interaction as this communication, either by direct control of brain waves or indirectly through muscle movements. Each of these methods, with different characteristics, provides an area of opportunity in pattern recognition, data fusion, accuracy of intention recognition, and development of less complex and more comfortable devices.

For a lower-limb prosthesis, the elements described in Section 3, HPI, sensing the external environment, electronics and digital systems, and control methods, must be considered at the same level of importance, but research must focus on improving them one at a time. Physical requirements such as weight, size, and materials are the first layer of interaction with a person, meaning the materials must be comfortable and durable. The same characteristics are directly related to the amount of energy used by an external power source and user effort, meaning engineers must take care to keep them lower.

Interaction with the environment and methods of measuring the performance of a prosthetic device are still being tested. Variables such as the kinematics for each person can variate metabolic cost and energy consumption, disregarding the prosthesis’s composition and developing a generalised prosthetic device at a lower cost. As with interaction with the environment, the prosthesis depends on the level of autonomy and the place where the artificial limb will be used.

Researchers who read this paper in the future should rely on this work to follow a structured design focussing on characteristics that could help amputees improve their quality of life and the option to perform leisure and everyday activities. Researchers can determine what is missing in the field by focussing on the trends found in this paper and combining efforts of engineers and healthcare workers.
